# Lancifoliaine, a New Bisbenzylisoquinoline from the Bark of *Litsea lancifolia*

**DOI:** 10.3390/molecules16043119

**Published:** 2011-04-13

**Authors:** Syazreen Nadia Sulaiman, Mat Ropi Mukhtar, A Hamid A Hadi, Khalijah Awang, Hazrina Hazni, Azeana Zahari, Marc Litaudon, Kazumasa Zaima, Hiroshi Morita

**Affiliations:** 1 Centre for Natural Products and Drug Discovery, Department of Chemistry, Faculty of Science, University of Malaya, 50603 Kuala Lumpur, Malaysia; Email: syaz_ryn@yahoo.com.my (S.N.S.); ahamid@um.edu.my (A.H.A.H.); khalijah@um.edu.my (K.A.); hazrinahazni@um.edu.my (H.H.); ezianna@gmail.com (A.Z.); 2 Centre de Recherche de Gif, Institut de Chimie des Substances Naturelles, CNRS, 1, Avenue de la Terrasse, 91198 Gif-sur-Yvette Cedex, France; Email: marc.litaudon@icsn.cnrs-gif.fr (M.L.); 3 Faculty of Pharmaceutical Sciences, Hoshi University, Shinagawa-ku, Tokyo 142-8501, Japan; Email: moritah@hoshi.ac.jp (H.M.)

**Keywords:** bisbenzylisoquinoline, lancifoliaine, *N*-allyllaurolitsine, Lauraceae, vaso-relaxant activity

## Abstract

A new bisbenzylisoquinoline, lancifoliaine (**1**), together with seven known alkaloids – *N*-allyllaurolitsine (**2**), reticuline (**3**), actinodaphnine, norboldine, pallidine, cassythicine and boldine – were isolated from the stem bark of *Litsea lancifolia* (Lauraceae). In addition to that of lancifoliaine, complete ^13^C-NMR data of *N*-allyl-laurolitsine (**2**) was also reported. The alkaloidal structures were elucidated by means of high field 1D- and 2D-NMR IR, UV, and LCMS-IT-TOF spectral data. *N*-Allyllaurolitsine (**2**) showed a moderate vasorelaxant activity on isolated rat aorta.

## 1. Introduction

In continuation of our research on plants from the Lauraceae family, we have embarked a study on the CH_2_Cl_2_ extract of the stem bark of *Litsea lancifolia* (known locally as *Medang melukut* [1]). Lauraceae plants are known to be prolific producers of many interesting alkaloids such as the rare proaporphine-tryptamine dimers: phoebegrandines A-B [2] and (-)-phoebescortechiniine [3], and bisbenzylisoquinoline alkaloids: oxoperakensimines A-C [4] and 3',4'-dihydronorstephasubine [5]. 

**Figure 1:**
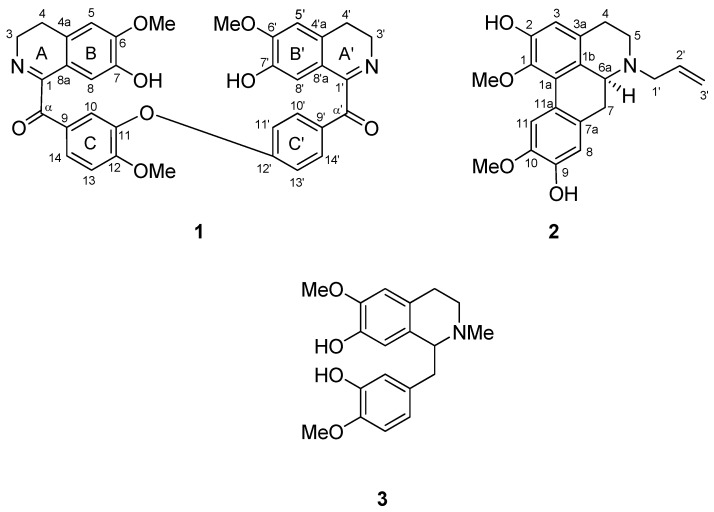
Structures of lancifoliaine (**1**), *N*-allyllaurolitsine (**2**) and reticuline (**3**).

The present study has led to the isolation of a new bisbenzylisoquinoline, lancifoliaine (**1**), together with *N*-allyllaurolitsine (**2**) [6], reticuline (**3**) [7,8,9], actinodaphnine [10], norboldine [11,12,13], pallidine [14,15,16], cassythicine [17] and boldine [18,19,20] (Figure 1).

## 2. Results and Discussion

Lancifoliaine (**1**) was isolated as a brown amorphous solid. The LCMS-IT-TOF spectrum of **1** showed a pseudomolecular ion peak, [M+H]^+^ at *m/z* 607.2183, corresponding to the molecular formula of C_35_H_31_N_2_O_8_. Absorption bands in the IR spectrum at 1,599 and 1,665 cm^−1^ were typical of an imine and carbonyl stretching bands [21]. In the ^1^H-NMR spectrum, signals for eleven aromatic protons due to three methoxy singlets and two –CH_2_-CH_2_-N- groups were observed, thus suggesting a bisbenzylisoquinoline type of skeleton [21,22]. Among the eleven aromatic proton signals, four singlets representing H-5, H-5', H-8 and H-8' appeared at δ 6.69, 6.71, 6.89 and 6.88 respectively. H-10 resonated as a doublet at δ 7.71 (*J* = 2.0 Hz) while H-14 appeared as a doublet of doublets at δ 7.95 (*J* = 8.8, 2.0 Hz) and H-13 exhibited as a doublet at δ 7.03 (*J* = 8.8 Hz), thus implying that ring C was trisubstituted. Ring C' showed signals of four aromatic protons; H-10' (dd, δ 7.95, *J *= 8.8, 2.0 Hz), H-14' (dd, δ 7.95, *J *= 8.8, 2.0 Hz), H-11' (d, δ 6.86, *J *= 8.8 Hz) and H-13' (d, δ 6.89, *J *= 8.8 Hz). This pattern indicating that it was a *para* disubstituted (AA'BB') ring system [23]. In addition, three methoxy groups appeared as singlets at δ 3.92 (6-OCH_3_), 3.84 (12-OCH_3_) and 3.91 (6'-OCH_3_). 

The ^13^C-NMR spectrum showed 35 carbon resonances, in agreement with the molecular formula. The presence of two carbonyl carbons was observed at δ 191.9 (C-α) and 192.7 (C-α'). The signals at δ165.1 and δ 164.9 could be assigned as the two imines C-1 and C-1' carbons, respectively. 

The position of ∆^1-N^ and ∆^1'-N'^ double bonds were confirmed by the HMBC correlation of H-8 to C-1 (δ 165.1) and correlation of H-8' to C-1' (δ 164.9). The most downfield signal at δ 162.6 was assignable to the oxygenated C-12' by the HMBC correlations of H-10' and H-14' (*J*_3_) to C-12' [24]. The presence of carbonyl groups at C-α and C-α' were also confirmed based on the HMBC correlation of H-10 (δ_H_ 7.71) to C-α (δ 191.9), and H-10' (δ 7.95) and H-14' (δ 7.95) to C-α' (δ 192.7) respectively. The ^1^H-NMR (400 MHz) and ^13^C-NMR (100 MHz) spectral assignments performed by extensive 2D NMR experiments (COSY, NOESY, HMQC and HMBC) were summarized in Table 1.

**Table 1:** ^1^H and ^13^C spectral data of lancifoliaine (**1**) in CDCl_3_.

Position	^1^H (δ_H_, *J*, Hz)	^13^C (δ_C_)	Position	^1^H (δ_H_, *J*, Hz)	^13^C (δ_C_)
1	-	165.1	1'	-	164.9
3	3.88, m	47.3	3'	3.88, m	47.3
4	2.76, m	25.5	4'	2.76, m	25.5
4a	-	130.2	4'a	-	130.2
5	6.69, s	110.1	5'	6.71, s	110.1
6	-	149.5	6'	-	149.4
6-OMe	3.92, s	56.3	6'-OMe	3.91, s	56.1
7	-	144.4	7'	-	144.4
8	6.89, s	113.1	8'	6.88, s	112.2
8a	-	119.9	8'a	-	119.9
α	-	191.9	α'	-	192.7
9	-	129.8	9'	-	129.8
10	7.71, d, *J* = 2.0 Hz	124.4	10'	7.95, dd, *J* = 8.8, 2.0 Hz	132.7
11	-	143.0	11'	6.86, d, *J* = 8.8 Hz	116.1
12	-	156.6	12'	-	162.6
12-OMe	3.84, s	56.3			
13	7.03, d, *J* = 8.8 Hz	112.2	13'	6.89, d, *J* = 8.8 Hz	116.1
14	7.95, dd, *J* = 8.8, 2.0 Hz	132.7	14'	7.95, dd, *J* = 8.8, 2.0 Hz	132.7

**Figure 2:**
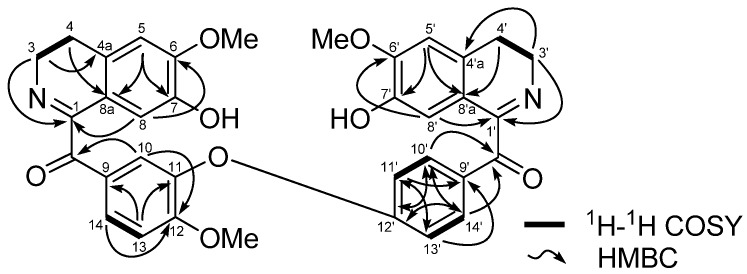
Selected 2D NMR correlations of lancifoliaine (**1**).

**Figure 3:**
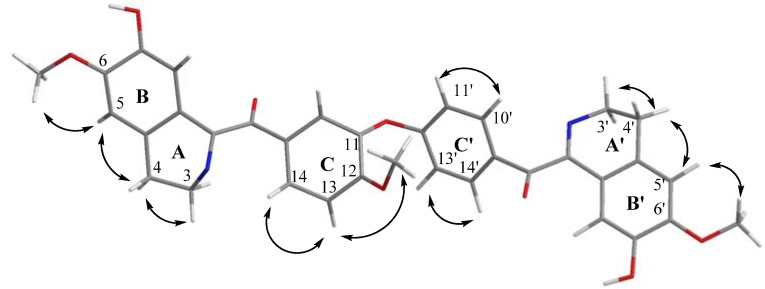
Selected NOESY correlations of lancifoliaine (**1**).

The COSY spectrum also showed cross-peaks between H-3/H-4, H-3'/H-4', H-10'/H-11', H-13/H-14 and also H-13'/H-14' (Figure 2). In addition, the position of the three methoxy groups, were assigned based on the NOESY cross-peaks between H-5/6-OCH_3_, H-5'/6'-OCH_3_ and H-13/12-OCH_3_ respectively. Selected NOESY correlations are shown in Figure 3.

*N*-Allyllaurolitsine (**2**), [α]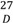 = +33.9º (*c* 1.0, MeOH) was isolated as a brownish amorphous solid. The LCMS-IT-TOF spectrum of **2** showed [M+H]^+^ peak at *m/z* 354.1823, corresponding to the molecular formula of C_21_H_24_NO_4_. ^1^H-NMR data of a synthetic compound of **2** were reported previously and we report herein the complete ^13^C-NMR assignments of **2**, which were established by thorough analysis of DEPT, HSQC and HMBC spectra [6]. This is the first communication on *N*-allyllaurolitsine as a natural compound and the ^13^C-NMR data is listed in Table 2.

**Table 2:** ^1^H and ^13^C spectral data of *N*-allyllaurolitsine (**2**) in CDCl_3_.

Position	^*1^H (δ_H_)	^13^C (δ_C_)
1	-	142.2
1a	-	126.3
1b	-	127.3
1-OMe	3.56, s	60.3
2	-	148.2
3	-	113.3
3a	-	130.4
4	-	28.8
5	-	49.1
6a	-	59.7
7	-	33.9
7a	-	130.4
8	6.81, s	114.2
9	-	145.2
10	-	145.8
10-OMe	3.90, s	56.2
11	7.86, s	110.1
11a	-	123.8
1' (N-C**H_2_**CH=CH_2_)	3.05, br d, *J* = 6.6 Hz	57.3
2' (N-CH_2_C**H**=CH_2_)	5.96, ddt, *J* = 17.2, 10.1, 6.6 Hz	134.3
3'		
(N-CH_2_CH=C**H_E_** H_Z_)	5.26, br d, *J* = 17.2 Hz	118.5
(N-CH_2_CH=CH_E_**H_Z_**)	5.19, br d, *J* = 10.1 Hz	

* ^1^H-NMR data are reproduced from Chiou *et al*. [6]

**Figure 4:**
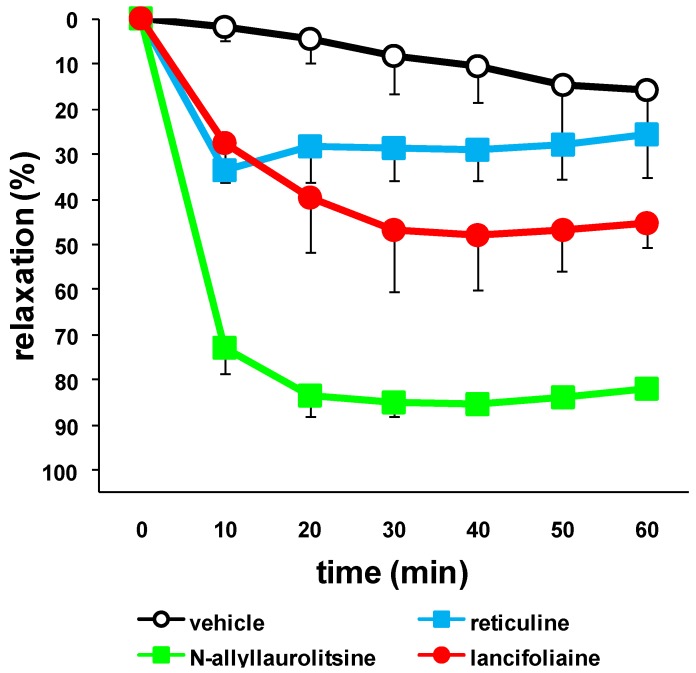
Relaxation responses induced by lancifoliaine (**1**; 10^−4^M), *N*-allyllaurolitsine (**2**; 10^−4^ M), and reticuline (**3**; 10^−4^ M) in aortic rings precontracted with 3 × 10^−7^ M phenylephrine (PE). Values are the mean ± S.E. (n = 3).

Vasodilators are useful for treatment of cerebral vasospasm and hypertension, and for improvement of peripheral circulation [25]. When phenylephrine (PE) 3 × 10^−7^ M was applied to thoracic aortic rings with endothelium after achieving a maximal response, we added lancifoliaine (**1**), *N*-allyllaurolitsine (**2**), and reticuline (**3**) as a related benzylisoquinoline alkaloid. *N*-Allyllaurolitsine (**2**) only showed a moderate vasorelaxant activity on isolated rat aorta (85% relaxation at × 10^−4^ M), whereas lancifoliaine (**1**) and reticuline (**3**) did not show any significant vasorelaxant activity (30% relaxation at × 10^−4^ M), as shown in Figure 4. Vasodilation seems to be influenced by substitution of a nitrogen atom. In the previous paper, we have reported vasorelaxant activities of some bisbenzylisoquinoline alkaloids such as α**ˊ**-oxoperakensimines A-C from *Alseodaphne perakensis* and *Alseodaphne corneri* [4,5]. Vasodilation may seem to be influenced by the asymmetric chirality of C-1. The mode of action of *N*-allyllaurolitsine (**2**) on vasorelaxant activity is under investigation.

## 3. Experimental

### 3.1. General

Spectra were recorded on the following instruments: UV, Shimadzu UV-250 UV-Visible spectrophotometer; IR, Perkin Elmer 1600; NMR, JEOL ECA 400 MHz; LCMS-IT-TOF, Shimadzu. All solvents, except those used for bulk extraction are AR grade. Silica gel 60 F254 was used for column chromatography. Glass and aluminium supported silica gel 60 F254 plates were used for preparative TLC. TLC spots were visualized under UV light (254 and 365 nm) followed by spraying with Dragendorff’s reagent for alkaloid detection.

### 3.2. Plant material

The bark of *Litsea lancifolia* was collected at Hutan Simpan Tembat, Ulu Terengganu (Malaysia) by the phytochemical group of the Department of Chemistry, Faculty of Science, University of Malaya. The voucher specimen (KL5208) has been deposited at the Herbarium of the Department of Chemistry, University of Malaya, Kuala Lumpur, Malaysia.

### 3.3. Extraction and Isolation

Dried, grounded bark of the plant (2.0 kg) was first defatted with hexane (16 L) twice for 3-days period. The hexane extract were first taken up to dryness. The plant material was dried up then soaked with 25% NH_4_OH (1 L) for 2 hours. It was then macerated with CH_2_Cl_2_ (16 L) twice for 3-days periods. The supernatant obtained was concentrated using a rotary evaporator under reduced pressure to a volume of 500 mL and were examined for their alkaloid content (using TLC and confirmed by spraying with Dragendorff’s reagent). The extract was finally concentrated to give crude alkaloids (8.0 g). The crude alkaloid (4.0 g) was subjected to column chromatography over silica gel using CH_2_Cl_2_ and methanol solvent (100:0, 99:1, 98:2, 95:5, and 90:10) and finally with 100% methanol was used as eluent to obtain twelve fractions. Further purification of fraction eight by a Preparative Thin Layer Chromatography (PTLC) yielded lancifoliaine (**1**, 15 mg, 98:2: saturated with NH_4_OH) and *N*-allyllaurolitsine (**2**, 25 mg, 98:2: saturated with NH_4_OH). 

*Lancifoliaine* (**1**). Brown amorphous solid, LCMS-IT-TOF at *m/z* 607.2183 ([M+H]^+^; calcd. for C_35_H_31_N_2_O_8_, 607.2080); UV (MeOH) 256 and 310 nm; IR (CHCl_3_) λ_max_: 3583, 3350, 2929, 1665 and 1599 cm^−1^; ^1^H and ^13^C-NMR: see Table 1.

*N*-Allyllaurolitsine (**2**). Brown amorphous solid, [α]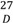 = +33.9º (c=1.0, MeOH), LCMS-IT-TOF at *m/z* 354.1823 ([M+H]^+^; calcd. for C_21_H_24_NO_4_, 354.1705); UV (MeOH) 307 nm; IR (CHCl_3_) λ_max_: 3584, 3372, 2955, 2352 and 1652 cm^−1^; ^1^H and ^13^C-NMR: see Table 2.

### 3.4. Vasodilation Assay

A male Wistar rat weighting 260 g was sacrificed by bleeding from the carotid arteries under anesthetization. A section of the thoracic aorta between the aortic arch and the diaphragm was removed and placed in oxygenated, modified Krebs-Henseleit solution (KHS: 118.0 mM NaCl, 4.7 mM KCl, 25.0 mM NaHCO_3_, 1.8 mM CaCl_2_, 1.2 mM NaH_2_PO_4_, 1.2 mM MgSO_4_, and 11.0 mM glucose). The aorta was cleaned of loosely adhering fat and connective tissue and cut into ring preparations 3 mm in length. The tissue was placed in a well-oxygenated (95% O_2_, 5% CO_2_) bath of 5 mL KHS solution at 37 °C with one end connected to a tissue holder and the other to a force-displacement transducer (Nihon Kohden, TB-611T). The tissue was equilibrated for 60 min under a resting tension of 1.0 g. During this time the KHS in the tissue bath was replaced every 20 min.

After equilibration, each aortic ring was contracted by treatment with 3 × 10^−7^ M PE. The presence of functional endothelial cells was confirmed by demonstrating relaxation to 10^−5^ M acetylcholine (ACh), and aortic ring in which 80% relaxation occurred, were regarded as tissues with endothelium. When the PE-induced contraction reached a plateau, each sample (**1**-**3**, ×10^−4^) was added.

These animal experimental studies were conducted in accordance with the Guiding Principles for the Care and Use of Laboratory Animals, Hoshi University and under the supervision of the Committee on Animal Research of Hoshi University, which is accredited by the Ministry of Education, Science, Sports Culture, and Technology of Japan.

## 4. Conclusions

Bisbenzylisoquinoline-type alkaloids with varied biological activities such as antiplasmodial, antibacterial, hypotensive, antitumor and anti-inflammatory effects have been reported to occur in various genera of the family of Lauraceae [4,5,26,27,28,29]. To our knowledge, this is the first report on the occurrence of bisbenzylisoquinoline alkaloid in the species *Litsea*. In fact, this is the second report on bisbenzylisoquinoline alkaloid with both *α* and *α'* positions oxidized forming carbonyl groups. The first related compound has been reported previously as a synthetic compound, 1,1'-[oxybis(p-phenylenecarbonyl)]bis[3,4-dihydro-6,7-dimethoxyisoquinoline] [30]. 

## References

[1] Whitmore T.C. (1972). Tree Flora of Malaya-A Manual for Foresters.

[2] Mukhtar M.R., Martin M.T., Domansky M., Pais M., Awang K., Hadi A.H.A. (1997). Phoebegrandines A and B, proaporphine-tryptamine dimers, from *Phoebe grandis*. Phytochemistry.

[3] Awang K., Mukhtar M.R., Mustafa M.R., Litaudon M., Shaari K., Mohamad K., Hadi A.H.A. (2007). New Alkaloids from *Phoebe scortechinii*. Nat. Prod. Res..

[4] Mukhtar M.R., Nafiah M.A., Awang K., Zaima K., Morita H., Hadi A.H.A. (2009). α*’*-Oxoperakensimines A – C, New Bisbenzylisoquinoline from *Alseodaphne perakensis* (Gamble) Kosterm. Heterocycles.

[5] Mukhtar M.R., Zahari A., Nafiah M.A., Hadi A.H.A., Thomas N.F., Arai H., Morita H., Litaudon M., Awang K. (2009). 3’,4’-Dihydronorstephasubine, A New Bisbenzylisoquinoline from the Bark of *Alseodaphne corneri*. Heterocycles.

[6] Chiou C.M., Kang J.J., Lee S.S. (1998). Litebamine *N*-Homologues: Preparation and Anti-Acetylcholinesterase Activity. J. Nat. Prod..

[7] Cava M.P., Rao K.V., Douglas B., Weisbach J.A. (1968). Alkaloids of *Cassytha americana*. J. Org. Chem..

[8] Oscar Castro C., José López V., Armando Vergara G. (1985). Aporphine Alkaloids from *Phoebe pittieri*. Phytochemistry.

[9] Janssen R.H.A.M., Wijkens P., Kruk C., Biessels H.W.A., Menichini F., Theuns H.G. (1990). Assignments of ^1^H and ^13^C NMR Resonances of Some Isoquinoline Alkaloids. Phytochemistry.

[10] Guinaudeau H., Leboeuf M., Cavé A. (1979). Aporphine Alkaloids. II. J. Nat. Prod..

[11] Tewari S., Bhakuni D.S., Dhar M.M. (1972). The Aporphine Alkaloids of *Litsea glutenosa*. Phytochemistry.

[12] Kozuka M., Miyazawa S., Yokoyama K., Odani T., Kubo M. (1985). Alkaloids from *Lindera umbellata*, *Lindera sericea*, and Their Varieties. J. Nat. Prod..

[13] Ross S.A., Minard R.D., Shamma M., Fagbule M.O., Olatunji G., Gbile Z. (1985). Thaliporphinemethine: A New Phenanthrene Alkaloid from *Illigera pentaphylla*. J. Nat. Prod..

[14] Guinaudeau H., Leboeuf M., Cavé A. (1983). Aporphinoid Alkaloids, III. J. Nat. Prod..

[15] Shamma M., Chinnasamy P., Hussain S.F., Khan F. (1976). Norpallidine, A New Morphinandienone Alkaloid from *Fumaria vaillantii*. Phytochemistry.

[16] Tojo E., Dominguez D., Castedo L. (1989). O-Methylpallidine N-oxide, the First Morphinandienone N-oxide Alkaloid. J. Nat. Prod..

[17] Gözler B., Öziç P., Freyer A.J., Shamma M. (1990). Morphinandienone Alkaloids from *Roemeria refracta*. J. Nat. Prod..

[18] Schiff P.L. (1991). Bisbenzylisoquinoline Alkaloids. J. Nat. Prod..

[19] Uprety H., Bhakuni D.S., Dhar M.M. (1972). Aporphine Alkaloids of *Litsea sebifera, L. wightiana* and *Actinodaphne obovata*. Phytochemistry.

[20] Leboeuf M., Caré A., Tohami M.E., Pusset J., Forgacs P., Provost J. (1982). Alkaloids of Annonaceae. XXXV. Alkaloids of *Desmos tiebaghiensis*. J. Nat. Prod..

[21] Chowdhury B.K., Sethi M.L., Lloyd H.A., Kapadia G.J. (1976). Aporphine and Tetrahydrobenzylisoquinoline Alkaloids in *Sassafras albidum*. Phytochemistry.

[22] Patra A., Freyer A.J., Guinaudeau H., Shamma M., Tantisewie B., Pharadai K. (1986). The Bisbenzylisoquinoline Alkaloids of *Stephania suberosa*. J. Nat. Prod..

[23] Diego C., Henry D., Rose L.R.P., Alaide B.O. (1987). Nouveaux Alcaloides Bis-benzylisoquinoleiques Isoles des Feuilles de *Aristolochia gigantean*. J. Nat. Prod..

[24] Pudjiastuti P., Mukhtar M.R., Hadi A.H.A., Saidi N., Morita H., Litaudon M., Awang K. (2010). (6,7-Dimethoxy-4-methylisoquinolinyl)-(4’-methoxyphenyl)-methanone, A New Benzylisoquinoline Alkaloid from *Beilschmiedia brevipes*. Molecules.

[25] Morita H., Iizuka T., Choo C.Y., Chan K.L., Takeya K., Kobayashi J. (2006). Vasorelaxant Activity of Cyclic Peptide, Cyclosquamosin B, from *Annona squamosa*. Bioorg. Med. Chem. Lett..

[26] Likhitwitayawaid K., Angerhofer C.K., Cordell G.A., Pezzuto J.M., Ruangrungsi N. (1993). Cytotoxic and Antimalarial Bisbenzylisoquinoline Alkaloids from *Stephania erecta*. J. Nat. Prod..

[27] Ke J., Weng S.A., Zhang G.Q. (1981). Effects of Tetrandrine on Experimental Arrhythmias. Acta Pharmacol. Sin..

[28] Li N., Li Y. (1986). Effects of Berbamine on Isolated Myocardium in Guinea Pigs and Humans. Acta Pharmacol. Sin..

[29] Seow W.K., Ferrante A., Goh D.B.H., Chalmers A.H., Li S., Thong Y.H. (1988). *In vitro* Immunosuppressive Properties of the Plant Alkaloid Tetrandrine. Int. Arch Allergy Apopl Immunol..

[30] Marini-Bettolo G.B., Chiavarelli S. (1952). Synthesis of bisquinolines derivatives on the model of natural alkaloids. I. Isoquinoline derivatives of diphenyl ether. Ann. Ist. Super. Sanita.

